# Supplementary material for: A Multi-Layered Study on Harmonic Oscillations in Mammalian Genomics and Proteomics
Source: Int J Mol Sci. 2019 Sep 17;20(18):4585. doi: 10.3390/ijms20184585 (PMC6770795; doi:10.3390/ijms20184585)
Supplement: Supplementary file 1 [file ijms-20-04585-s001.zip › FigureS1.pdf]

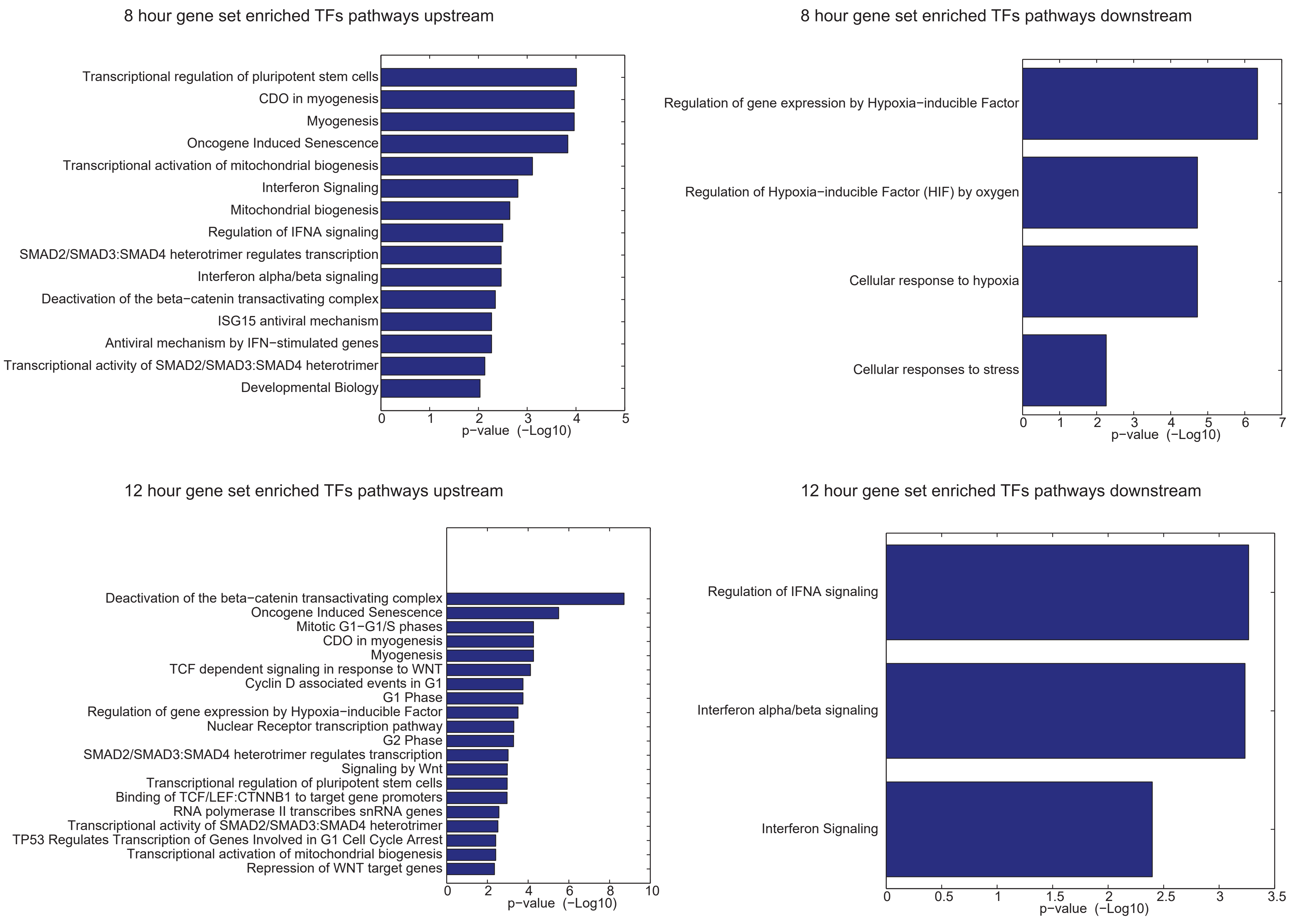
8 hour gene set enriched TFs pathways upstream
Transcriptional regulation of pluripotent stem cells
CDO in myogenesis
Myogenesis
Oncogene Induced Senescence
Transcriptional activation of mitochondrial biogenesis
Interferon Signaling
Mitochondrial biogenesis
Regulation of IFNA signaling
SMAD2/SMAD3:SMAD4 heterotrimer regulates transcription
Interferon alpha/beta signaling
Deactivation of the beta−catenin transactivating complex
ISG15 antiviral mechanism
Antiviral mechanism by IFN−stimulated genes
Transcriptional activity of SMAD2/SMAD3:SMAD4 heterotrimer
Developmental Biology
0 1 2 3 4 5
p−value (−Log10)
8 hour gene set enriched TFs pathways downstream
Regulation of gene expression by Hypoxia−inducible Factor
Regulation of Hypoxia−inducible Factor (HIF) by oxygen
Cellular response to hypoxia
Cellular responses to stress
0 1 2 3 4 5 6 7
p−value (−Log10)
12 hour gene set enriched TFs pathways upstream
Deactivation of the beta−catenin transactivating complex
Oncogene Induced Senescence
Mitotic G1−G1/S phases
CDO in myogenesis
Myogenesis
TCF dependent signaling in response to WNT
Cyclin D associated events in G1
G1 Phase
Regulation of gene expression by Hypoxia−inducible Factor
Nuclear Receptor transcription pathway
G2 Phase
SMAD2/SMAD3:SMAD4 heterotrimer regulates transcription
Signaling by Wnt
Transcriptional regulation of pluripotent stem cells
Binding of TCF/LEF:CTNNB1 to target gene promoters
RNA polymerase II transcribes snRNA genes
Transcriptional activity of SMAD2/SMAD3:SMAD4 heterotrimer
TP53 Regulates Transcription of Genes Involved in G1 Cell Cycle Arrest
Transcriptional activation of mitochondrial biogenesis
Repression of WNT target genes
0 2 4 6 8 10
p−value (−Log10)
12 hour gene set enriched TFs pathways downstream
Regulation of IFNA signaling
Interferon alpha/beta signaling
Interferon Signaling
0 0.5 1 1.5 2 2.5 3 3.5
p−value (−Log10)
