# Supplementary material for: A Multi-Layered Study on Harmonic Oscillations in Mammalian Genomics and Proteomics
Source: Int J Mol Sci. 2019 Sep 17;20(18):4585. doi: 10.3390/ijms20184585 (PMC6770795; doi:10.3390/ijms20184585)
Supplement: Supplementary file 1 [file ijms-20-04585-s001.zip › FigureS3.pdf]

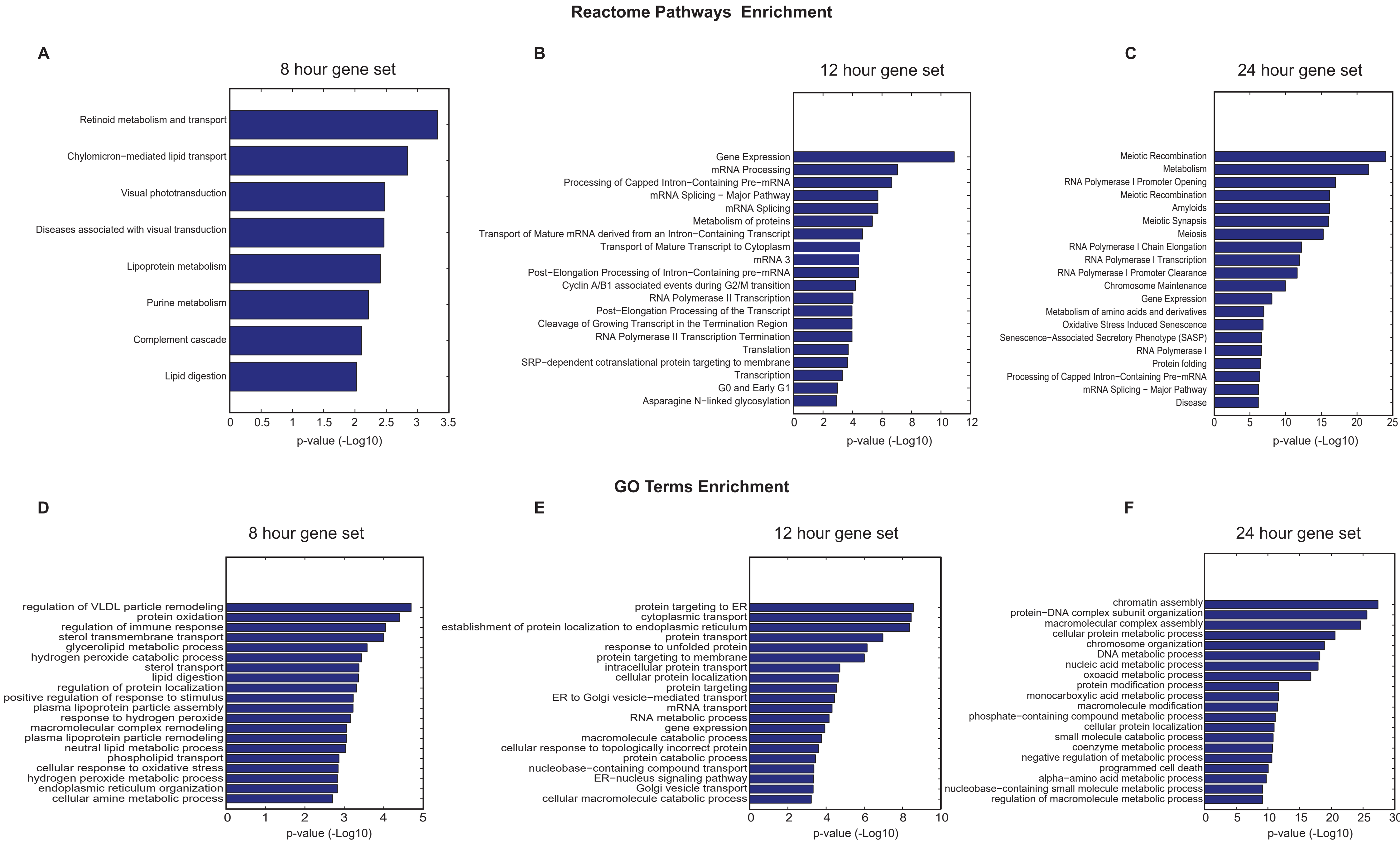
Reactome Pathways Enrichment
A
8 hour gene set
Retinoid metabolism and transport
Chylomicron−mediated lipid transport
Visual phototransduction
Diseases associated with visual transduction
Lipoprotein metabolism
Purine metabolism
Complement cascade
Lipid digestion
0 0.5 1 1.5 2 2.5 3 3.5
p-value (-Log10)
B
12 hour gene set
Gene Expression
mRNA Processing
Processing of Capped Intron−Containing Pre−mRNA
mRNA Splicing − Major Pathway
mRNA Splicing
Metabolism of proteins
Transport of Mature mRNA derived from an Intron−Containing Transcript
Transport of Mature Transcript to Cytoplasm
mRNA 3
Post−Elongation Processing of Intron−Containing pre−mRNA
Cyclin A/B1 associated events during G2/M transition
RNA Polymerase II Transcription
Post−Elongation Processing of the Transcript
Cleavage of Growing Transcript in the Termination Region
RNA Polymerase II Transcription Termination
Translation
SRP−dependent cotranslational protein targeting to membrane
Transcription
G0 and Early G1
Asparagine N−linked glycosylation
0 2 4 6 8 10 12
p-value (-Log10)
C
24 hour gene set
Meiotic Recombination
Metabolism
RNA Polymerase I Promoter Opening
Meiotic Recombination
Amyloids
Meiotic Synapsis
Meiosis
RNA Polymerase I Chain Elongation
RNA Polymerase I Transcription
RNA Polymerase I Promoter Clearance
Chromosome Maintenance
Gene Expression
Metabolism of amino acids and derivatives
Oxidative Stress Induced Senescence
Senescence−Associated Secretory Phenotype (SASP)
RNA Polymerase I
Protein folding
Processing of Capped Intron−Containing Pre−mRNA
mRNA Splicing − Major Pathway
Disease
0 5 10 15 20 25
p-value (-Log10)
GO Terms Enrichment
D
8 hour gene set
regulation of VLDL particle remodeling
protein oxidation
regulation of immune response
sterol transmembrane transport
glycerolipid metabolic process
hydrogen peroxide catabolic process
sterol transport
lipid digestion
regulation of protein localization
positive regulation of response to stimulus
plasma lipoprotein particle assembly
response to hydrogen peroxide
macromolecular complex remodeling
plasma lipoprotein particle remodeling
neutral lipid metabolic process
phospholipid transport
cellular response to oxidative stress
hydrogen peroxide metabolic process
endoplasmic reticulum organization
cellular amine metabolic process
0 1 2 3 4 5
p-value (-Log10)
E
12 hour gene set
protein targeting to ER
cytoplasmic transport
establishment of protein localization to endoplasmic reticulum
protein transport
response to unfolded protein
protein targeting to membrane
intracellular protein transport
cellular protein localization
protein targeting
ER to Golgi vesicle−mediated transport
mRNA transport
RNA metabolic process
gene expression
macromolecule catabolic process
cellular response to topologically incorrect protein
protein catabolic process
nucleobase−containing compound transport
ER−nucleus signaling pathway
Golgi vesicle transport
cellular macromolecule catabolic process
0 2 4 6 8 10
p-value (-Log10)
F
24 hour gene set
chromatin assembly
protein−DNA complex subunit organization
macromolecular complex assembly
cellular protein metabolic process
chromosome organization
DNA metabolic process
nucleic acid metabolic process
oxoacid metabolic process
protein modification process
monocarboxylic acid metabolic process
macromolecule modification
phosphate−containing compound metabolic process
cellular protein localization
small molecule catabolic process
coenzyme metabolic process
negative regulation of metabolic process
programmed cell death
alpha−amino acid metabolic process
nucleobase−containing small molecule metabolic process
regulation of macromolecule metabolic process
0 5 10 15 20 25 30
p-value (-Log10)
