# Supplementary material for: A Multi-Layered Study on Harmonic Oscillations in Mammalian Genomics and Proteomics
Source: Int J Mol Sci. 2019 Sep 17;20(18):4585. doi: 10.3390/ijms20184585 (PMC6770795; doi:10.3390/ijms20184585)

The histone modification of the oscillating genes is strongly tissue specific with enrichment of the H3K79m2 methylation of the 12h and 24h gene sets

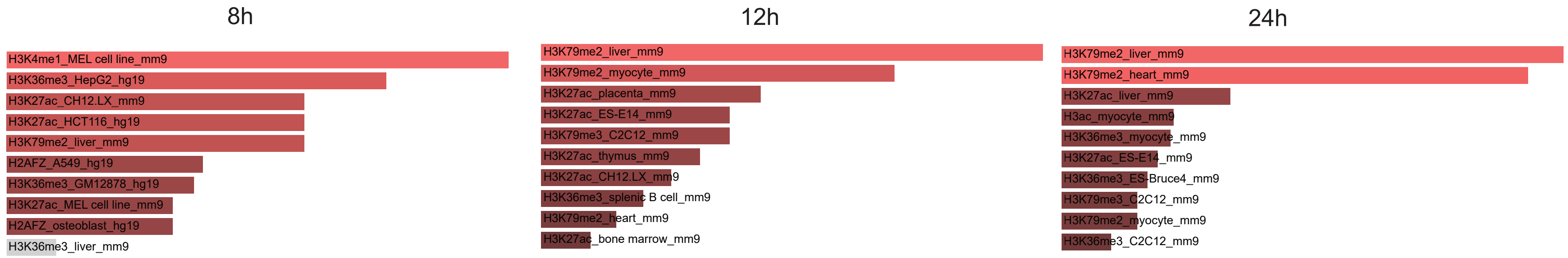

The RNA Polymerase II plays a major role in the transcription regulation of the 12h and 24h oscillating genes as previously suggested by the H3K79me2

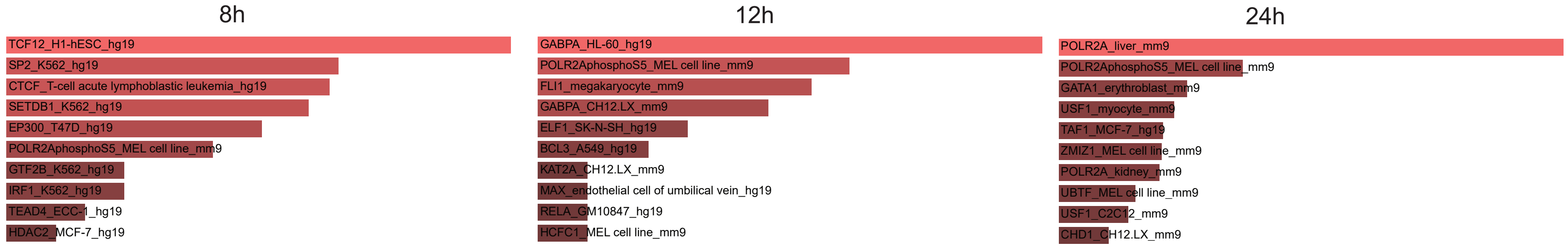

Computationally determined targets for the miRNA 1295 are strongly enriched in the 8 hour gene set

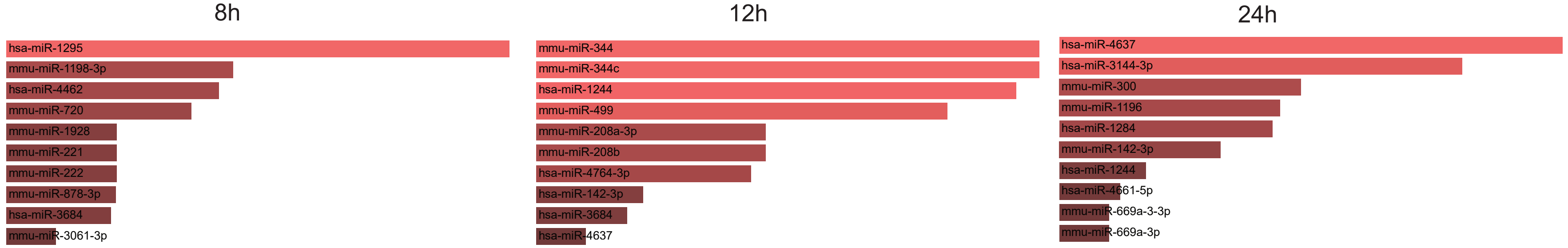

The protein-protein-interactions with TFs show a clear enrichment of the POLE TF for the 8 hour gene set and ESR1 TF for the 12h and 24h gene sets

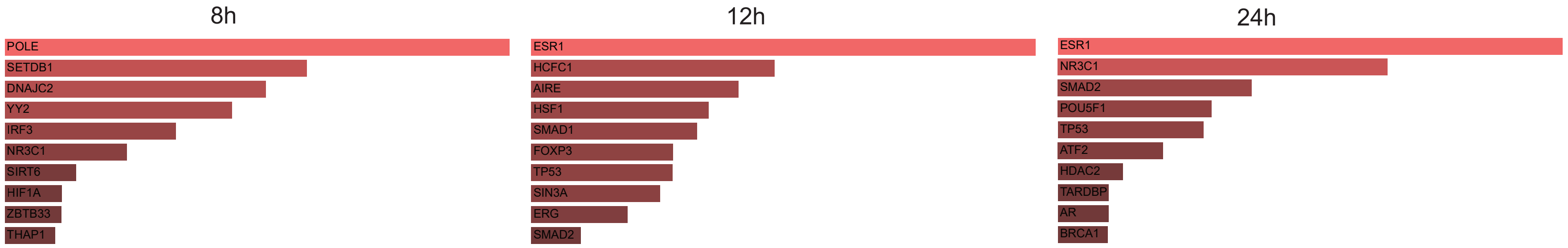

Supplement: Supplementary file 1 [file ijms-20-04585-s001.zip › FigureS4.pdf]
