# Supplementary material for: A Multi-Layered Study on Harmonic Oscillations in Mammalian Genomics and Proteomics
Source: Int J Mol Sci. 2019 Sep 17;20(18):4585. doi: 10.3390/ijms20184585 (PMC6770795; doi:10.3390/ijms20184585)
Supplement: Supplementary file 1 [file ijms-20-04585-s001.zip › SupplementaryMaterials_Legends.pdf]

Legends for Supplementary Materials:

Figure S1. Enrichment analysis for Gene Ontology and Reactome Pathways terms in the transcription factors for which enriched binding sites were detected in the promoter regions of the 8-hours, 12-hours and 24-hours gene sets.

Figure S2. High-resolution format of Figure 2.

Figure S3. Pathways and GO terms enrichment analysis points at the specific functions for each gene set. Enrichment analysis was performed both for Reactome pathways (A, B, C) and for GO terms (D, E, F). Each of the three sets of oscillating genes was analysed individually.

Figure S4. Enrichment analysis of histone modifications. A) Enrichment analysis of histone modifications associated with the gene sets show a preference for the H3K79me2 methylation in the 12-hours and 24-hours gene sets. Based on data from the encode project the H3K79me2 methylation is associated with the 12-hours and 24-hours gene sets. This methylation is connected to the activity of the RNA polymerase II and is highly tissue specific (liver). B) The RNA Polymerase II plays a major role in the regulation of the 12-hours and 24-hours gene sets. Based on ChIP-Seq data from the Encode project the activity of the RNA polymerase was associated with the 12-hours and 24-hours gene sets. However, other TFs such as TCF12 score higher in the 8-hours gene set. C) Targets for a single microRNA – miRNA 1295 dominate the enrichment for the 8-hours gene set: The enrichment analysis for computationally predicted miRNA targets from the TargetSCAN 2017 database delivers very strong results for the 24-hours and 8-hours gene sets. For the 8-hours gene set the targets for miRNA 1295 clearly are the dominant signal (p = 6.563e-03). Computationally determined targets for the miRNA 4637 are enriched in the 24-hours gene set (p = 1.630e-05) while in the 12-hours gene set four microRNA target sets dominate: miRNA 344 (p = 6.117e-04), miRNA 344c (p = 6.117e-04), miRNA 1244 (p = 7.081e-04) and miRNA 499 (p = 1.096e-03). D) Protein-Protein interaction data for the transcription factors associated with the 8-hours, 12-hours and 24-hours gene sets. Interactions associated with POLE are enriched for the 8-hours gene set and interactions associated with ESR1 are enriched for the 12-hours and 24-hours gene sets.

Figure S5. High POLE expression is associated with negative outcome for renal cancer and melanoma.

Figure S6. Correlation matrix for parameters of the 8-hours, 12-hours and 24-hours gene sets. A) Correlation matrix for parameters of genes with oscillating expression levels with a period of 8 hours. Numbers in the boxes are Spearman's correlation coefficients; asterisks represent statistical significant correlations (* $p < 0.05$, ** $p < 0.01$, *** $p < 0.001$). B) Correlation matrix for parameters of genes with oscillating expression levels with a period of 12 hours. Numbers in the boxes are Spearman's correlation coefficients; asterisks represent statistical significant correlations (* $p < 0.05$, ** $p < 0.01$, *** $p < 0.001$). C) Correlation matrix for parameters of oscillating genes with a period of 24 hours. Numbers in the boxes are Spearman's correlation coefficients; asterisks represent statistical significant correlations (* $p < 0.05$, ** $p < 0.01$, *** $p < 0.001$).

Figure S7. Cytoscape files for the network in Figure 4.

Table S1. Enriched binding sites for known transcription factors in the upstream regions of the promoters of the 8-hour gene set.

Table S2. Enriched binding sites for known transcription factors in the downstream regions of the promoters of the 8-hour gene set.

Table S3. Enriched binding sites for known transcription factors in the upstream regions of the promoters of the 12-hour gene set.

Table S4. Enriched binding sites for known transcription factors in the downstream regions of the promoters of the 12-hour gene set.

Table S5. Enriched binding sites for known transcription factors in the downstream regions of the promoters of the 24-hour gene set.

Table S6. Enriched binding sites for known transcription factors in the upstream regions of the promoters of the 24-hour gene set.

Table S7. Descriptive statistics for the data in Table 1.

Table S8. Free energy (ΔG) terms included in the core function of FoldX, the empirical force field algorithm aiming to calculate the change of ΔG in kcalmol^{-1}.

Table S9. Chromosome mapping of oscillating genes.
