# Supplementary figures and images for: A Multi-Layered Study on Harmonic Oscillations in Mammalian Genomics and Proteomics
Source: Int J Mol Sci. 2019 Sep 17;20(18):4585. doi: 10.3390/ijms20184585 (PMC6770795; doi:10.3390/ijms20184585)

A

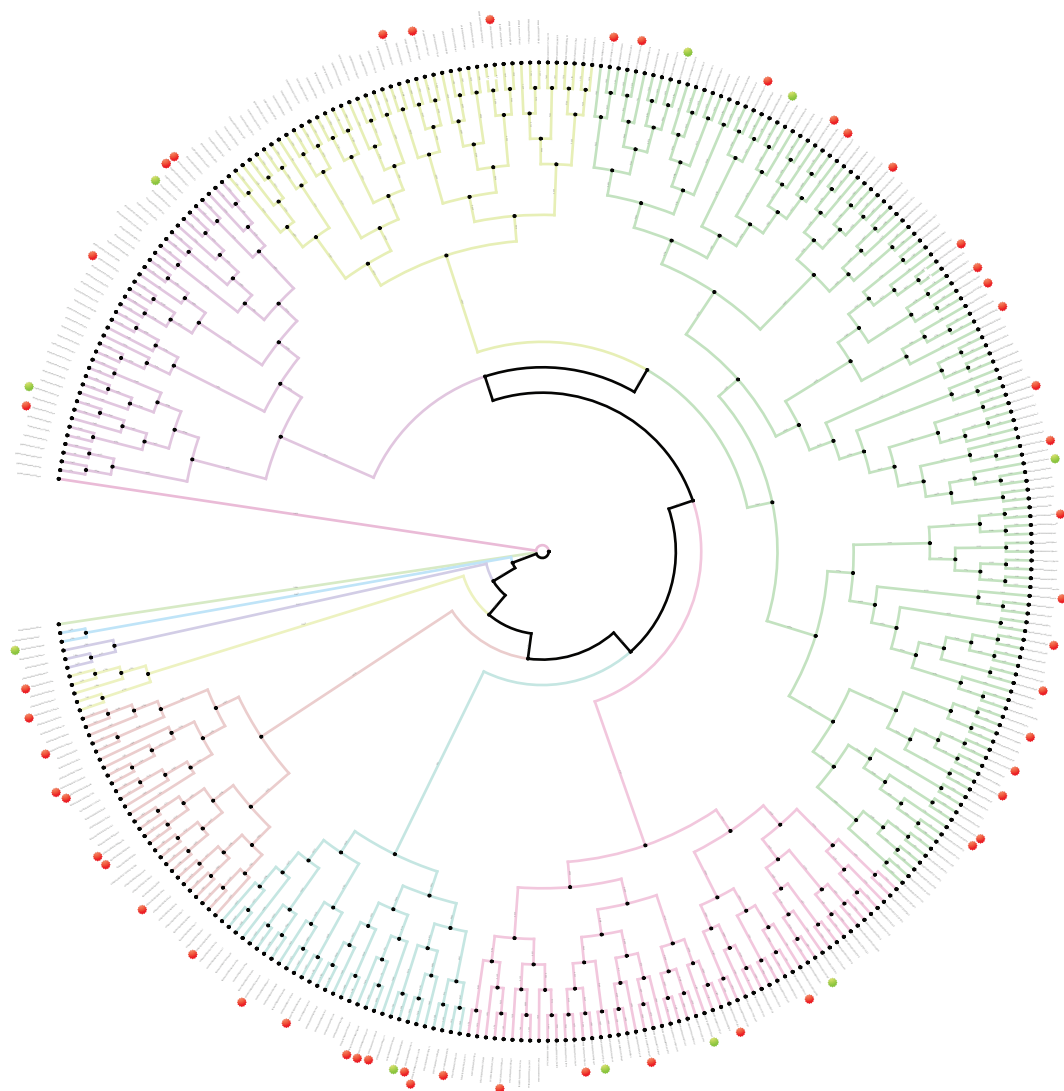

B

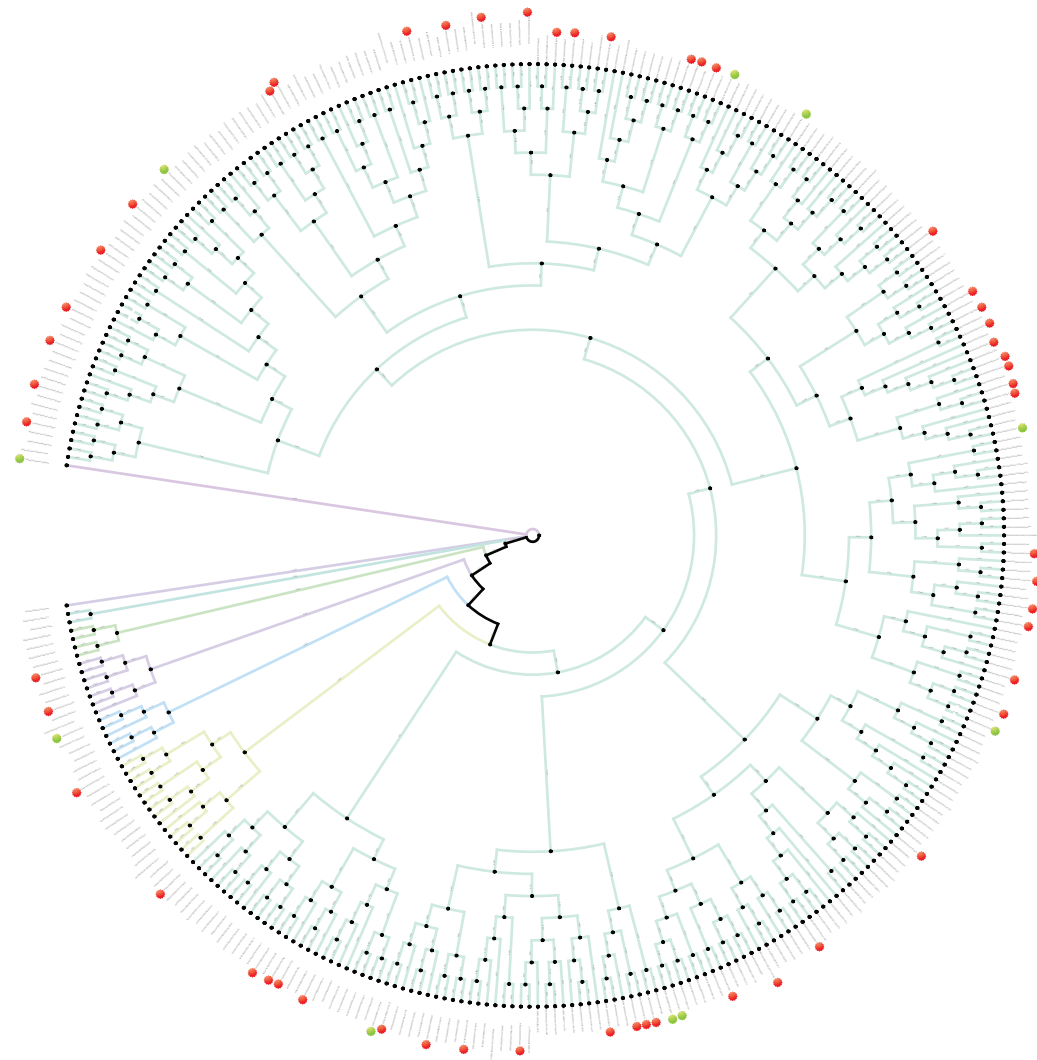

Supplement: Supplementary file 1 [file ijms-20-04585-s001.zip › FigureS2.pdf]

A

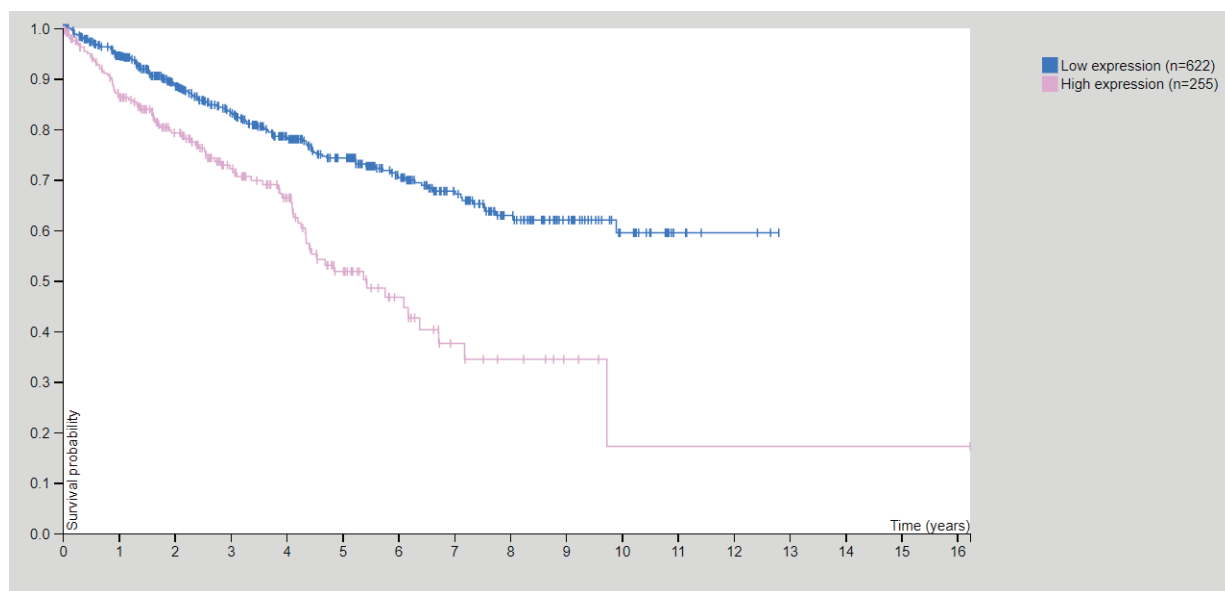

B

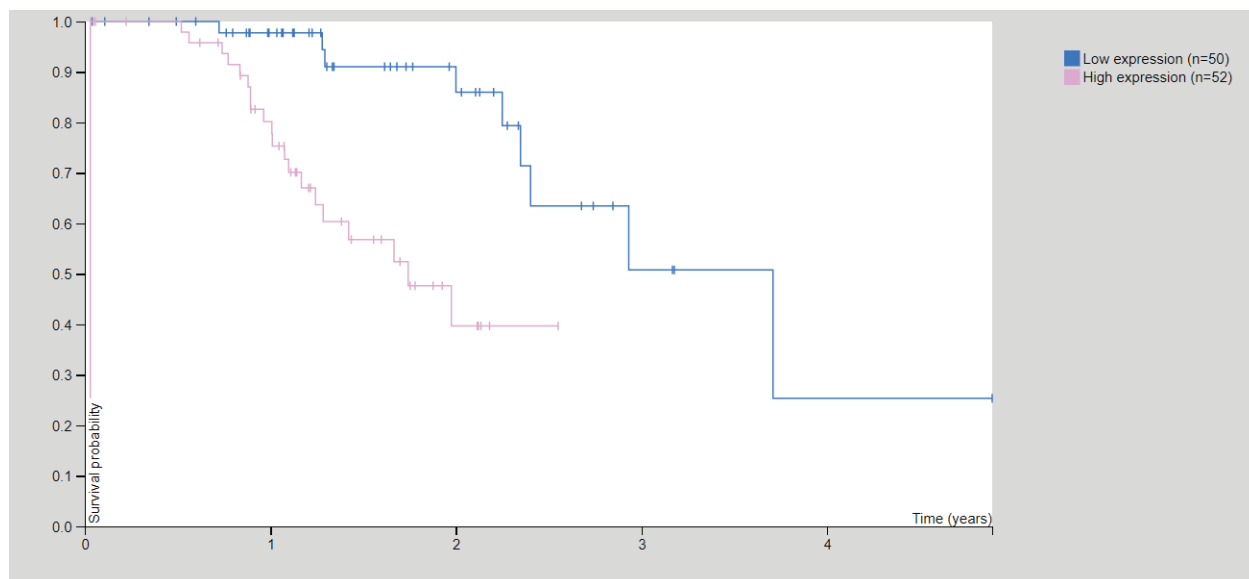

Supplement: Supplementary file 1 [file ijms-20-04585-s001.zip › FigureS5.pdf]

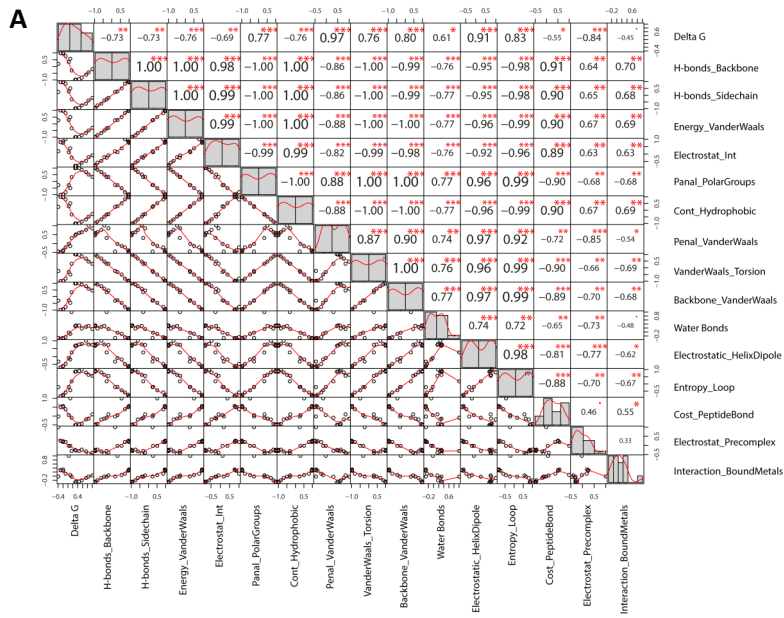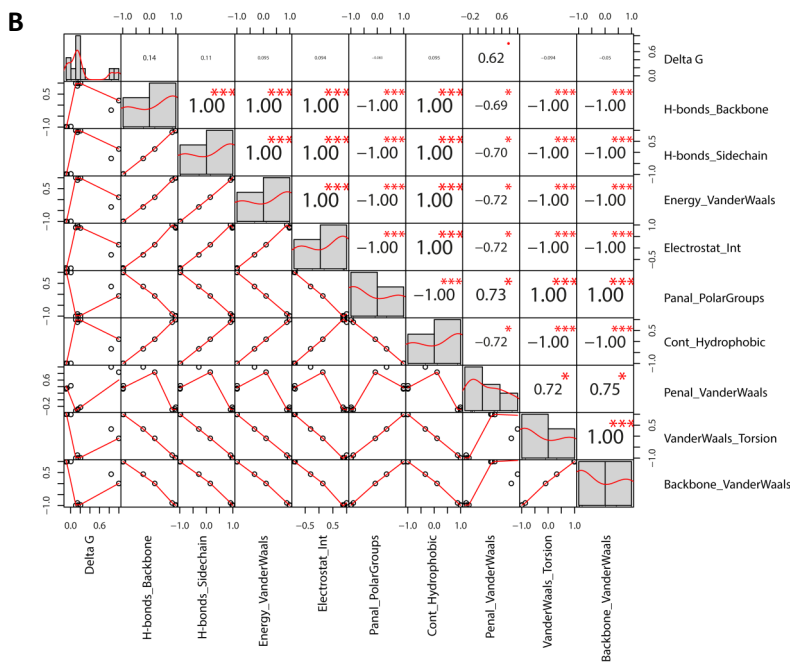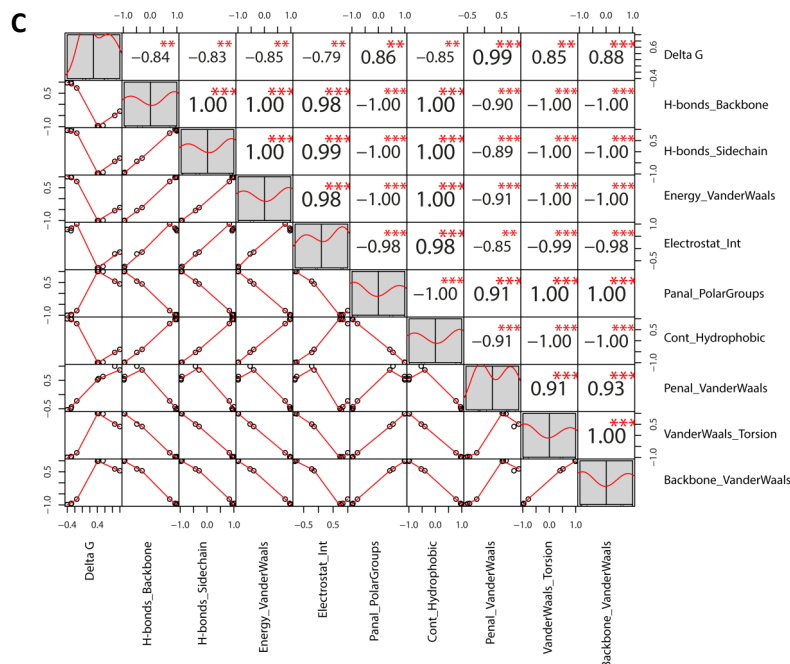

Supplement: Supplementary file 1 [file ijms-20-04585-s001.zip › FigureS6.pdf]
