# Supplementary material for: A Multi-Layered Study on Harmonic Oscillations in Mammalian Genomics and Proteomics
Source: Int J Mol Sci. 2019 Sep 17;20(18):4585. doi: 10.3390/ijms20184585 (PMC6770795; doi:10.3390/ijms20184585)
Supplement: Supplementary file 1 [file ijms-20-04585-s001.zip › TableS7.doc]

**Table S7 - Descriptive Statistics**

**8-hour oscillating genes**

| **Column** | **Size** | **Missing** | | **Mean** | | **Std Dev** | | **Std. Error** | | **C.I. of Mean** |
| --- | --- | --- | --- | --- | --- | --- | --- | --- | --- | --- |
| DG | 15 | 0 | | 65,943 | | 120,468 | | 31,105 | | 66,713 |
| Back bond | 15 | 0 | | -139,474 | | 89,966 | | 23,229 | | 49,821 |
| Side Hbond | 15 | 0 | | -66,112 | | 54,732 | | 14,132 | | 30,309 |
| Energy VdW | 15 | 0 | | -240,669 | | 160,300 | | 41,389 | | 88,771 |
| Electro | 15 | 0 | | -9,426 | | 10,277 | | 2,654 | | 5,691 |
| Energy SolvP | 15 | 0 | | 336,013 | | 223,528 | | 57,715 | | 123,786 |
| Energy SolvH | 15 | 0 | | -313,727 | | 208,663 | | 53,877 | | 115,554 |
| Energy vdwclas | 15 | 0 | | 16,201 | | 24,202 | | 6,249 | | 13,403 |
| energy torsion | 15 | 0 | | 132,476 | | 92,672 | | 23,928 | | 51,320 |
| backbone vdw | 15 | 0 | | 347,197 | | 220,820 | | 57,016 | | 122,286 |
| Entropy sidec | 15 | 0 | | 0,000 | | 0,000 | | 0,000 | | 0,000 |
| Entropy manc | 15 | 0 | | 0,000 | | 0,000 | | 0,000 | | 0,000 |
| water bonds | 15 | 0 | | 0,174 | | 0,397 | | 0,102 | | 0,220 |
| helix dipole | 15 | 0 | | 7,446 | | 7,047 | | 1,819 | | 3,902 |
| loop entropy | 15 | 2 | | 184,210 | | 126,705 | | 35,142 | | 76,567 |
| cis bond | 15 | 0 | | -3,040 | | 3,409 | | 0,880 | | 1,888 |
| disulfide | 15 | 0 | | 0,000 | | 0,000 | | 0,000 | | 0,000 |
| kn electrostatic | 15 | 0 | | 0,000 | | 0,000 | | 0,000 | | 0,000 |
| partial covalent | 15 | 0 | | 0,000 | | 0,000 | | 0,000 | | 0,000 |
| Energy Ionisation | 15 | 0 | | -1,234 | | 4,305 | | 1,111 | | 2,384 |
| Entropy Complex | 15 | 0 | | 0,634 | | 0,840 | | 0,217 | | 0,465 |
| Residue Number | 15 | 4 | | 224,000 | | 136,621 | | 41,193 | | 91,783 |
| **Column** | **Range** | | **Max** | | **Min** | | **Median** | |  |  |
| DG | 501,421 | | 406,573 | | -94,848 | | 59,387 | |  |  |
| Back bond | 294,067 | | -22,350 | | -316,417 | | -130,838 | |  |  |
| Side Hbond | 178,565 | | -8,767 | | -187,332 | | -46,078 | |  |  |
| Energy VdW | 513,324 | | -45,633 | | -558,957 | | -203,880 | |  |  |
| Electro | 32,165 | | -0,859 | | -33,024 | | -4,330 | |  |  |
| Energy SolvP | 706,594 | | 780,759 | | 74,165 | | 268,660 | |  |  |
| Energy SolvH | 658,286 | | -54,842 | | -713,128 | | -267,345 | |  |  |
| Energy vdwclas | 96,787 | | 98,421 | | 1,634 | | 9,525 | |  |  |
| Entropy sidec | 0 | | 0,000 | | 0,000 | | 0,000 | |  |  |
| Entropy manc | 0 | | 0,000 | | 0,000 | | 0,000 | |  |  |
| water bonds | 1,12406 | | 1,12406 | | 0,000 | | 0,000 | |  |  |
| helix dipole | 27,6289 | | 26,2052 | | 1,42369 | | 5,32356 | |  |  |
| loop entropy | 539,045 | | 484,813 | | 54,2323 | | 164,584 | |  |  |
| cis bond | 12,8183 | | 0,000 | | -12,8183 | | -2,55695 | |  |  |
| disulfide | 0 | | 0,000 | | 0,000 | | 0,000 | |  |  |
| kn electrostatic | 0 | | 0,000 | | 0,000 | | 0,000 | |  |  |
| partial covalent | 0 | | 0,000 | | 0,000 | | 0,000 | |  |  |
| Energy Ionisation | 16,5038 | | 1,07061 | | -154332 | | 0,000 | |  |  |
| Entropy Complex | 2,64794 | | 2,64794 | | 0,000 | | 0,18296 | |  |  |
| Residue Number | 553 | | 479 | | 74 | | 184 | |  |  |

**12-hour oscillating genes**

| **Column** | **Size** | **Missing** | | **Mean** | | **Std Dev** | | **Std. Error** | | **C.I. of Mean** | |
| --- | --- | --- | --- | --- | --- | --- | --- | --- | --- | --- | --- |
| DG | 30 | 0 | | 13,534 | | 95,472 | | 17,431 | | 35,650 | |
| Back bond | 30 | 0 | | -236,160 | | 157,462 | | 28,749 | | 58,797 | |
| Side Hbond | 30 | 0 | | -103,979 | | 65,354 | | 11,932 | | 24,404 | |
| Energy VdW | 30 | 0 | | -407,371 | | 260,653 | | 47,588 | | 97,329 | |
| Electro | 30 | 0 | | -16,011 | | 9,858 | | 1,800 | | 3,681 | |
| Energy SolvP | 30 | 0 | | 541,546 | | 339,781 | | 62,035 | | 126,876 | |
| Energy SolvH | 30 | 0 | | -542,341 | | 350,610 | | 64,012 | | 130,920 | |
| Energy vdwclas | 30 | 0 | | 22,333 | | 23,026 | | 4,204 | | 8,598 | |
| energy torsion | 30 | 0 | | 223,092 | | 151,230 | | 27,611 | | 56,470 | |
| backbone vdw | 30 | 0 | | 532,710 | | 331,294 | | 60,486 | | 123,707 | |
| Entropy sidec | 30 | 0 | | 0,000 | | 0,000 | | 0,000 | | 0,000 | |
| Entropy manc | 30 | 0 | | 0,000 | | 0,000 | | 0,000 | | 0,000 | |
| water bonds | 30 | 0 | | 0,550 | | 0,742 | | 0,136 | | 0,277 | |
| helix dipole | 30 | 0 | | 12,091 | | 9,241 | | 1,687 | | 3,450 | |
| loop entropy | 30 | 0 | | 240,492 | | 151,955 | | 27,743 | | 56,741 | |
| cis bond | 30 | 0 | | -5,705 | | 5,276 | | 0,963 | | 1,970 | |
| disulfide | 30 | 0 | | 0,000 | | 0,000 | | 0,000 | | 0,000 | |
| kn electrostatic | 30 | 0 | | 0,000 | | 0,000 | | 0,000 | | 0,000 | |
| partial covalent | 30 | 0 | | -0,0527 | | 0,288 | | 0,0527 | | 0,108 | |
| Energy Ionisation | 30 | 0 | | -8,547 | | 14,491 | | 2,646 | | 5,411 | |
| Entropy Complex | 30 | 0 | | 1,379 | | 1,024 | | 0,187 | | 0,382 | |
| Residue Number | 30 | 0 | | 373,267 | | 232,067 | | 42,369 | | 86,655 | |
| **Column** | **Range** | | **Max** | | **Min** | | **Median** | |  | |  |
| DG | 505,768 | | 387,860 | | -117,908 | | -3,947 | |  | |  |
| Back bond | 769,180 | | -35,960 | | -805,140 | | -194,457 | |  | |  |
| Side Hbond | 298,518 | | -20,376 | | -318,894 | | -94,556 | |  | |  |
| Energy VdW | 1238,654 | | -70,456 | | -1309,110 | | -376,421 | |  | |  |
| Electro | 32,394 | | -0,611 | | -33,005 | | -15,288 | |  | |  |
| Energy SolvP | 1590,950 | | 1685,830 | | 94,879 | | 512,976 | |  | |  |
| Energy SolvH | 1673,505 | | -93,765 | | -1767,270 | | -498,350 | |  | |  |
| Energy vdwclas | 110,051 | | 110,910 | | 0,859 | | 14,814 | |  | |  |
| energy torsion | 751,344 | | 793,016 | | 41,672 | | 209,531 | |  | |  |
| backbone vdw | 1575,469 | | 1676,280 | | 100,811 | | 488,894 | |  | |  |
| Entropy sidec | 0,000 | | 0,000 | | 0,000 | | 0,000 | |  | |  |
| Entropy manc | 0,000 | | 0,000 | | 0,000 | | 0,000 | |  | |  |
| water bonds | 2,248 | | 2,248 | | 0,000 | | 0,000 | |  | |  |
| helix dipole | 36,636 | | 38,210 | | 1,574 | | 8,613 | |  | |  |
| loop entropy | 726,807 | | 751,576 | | 24,769 | | 211,953 | |  | |  |
| cis bond | 18,945 | | 0,349 | | -18,596 | | -4,738 | |  | |  |
| disulfide | 0,000 | | 0,000 | | 0,000 | | 0,000 | |  | |  |
| kn electrostatic | 0,000 | | 0,000 | | 0,000 | | 0,000 | |  | |  |
| partial covalent | 1,580 | | 0,000 | | -1,580 | | 0,000 | |  | |  |
| Energy Ionisation | 37,428 | | 0,000 | | -37,428 | | 0,000 | |  | |  |
| Entropy Complex | 4,495 | | 4,495 | | 0,000 | | 1,179 | |  | |  |
| Residue Number | 1086,000 | | 1156,000 | | 70,000 | | 335,000 | |  | |  |

**24-hour oscillating genes**

| **Column** | **Size** | | **Missing** | | **Mean** | | **Std Dev** | | **Std. Error** | | **C.I. of Mean** | |
| --- | --- | --- | --- | --- | --- | --- | --- | --- | --- | --- | --- | --- |
| DG | 204 | | 0 | | 21,625 | | 125,741 | | 8,804 | | 17,358 | |
| Back bond | 204 | | 0 | | -206,118 | | 104,258 | | 7,299 | | 14,393 | |
| Side Hbond | 204 | | 0 | | -87,675 | | 45,528 | | 3,188 | | 6,285 | |
| Energy VdW | 204 | | 0 | | -354,538 | | 191,389 | | 13,400 | | 26,421 | |
| Electro | 204 | | 0 | | -13,710 | | 9,049 | | 0,634 | | 1,249 | |
| Energy SolvP | 204 | | 0 | | 472,585 | | 255,735 | | 17,905 | | 35,304 | |
| Energy SolvH | 204 | | 0 | | -470,759 | | 254,048 | | 17,787 | | 35,071 | |
| Energy vdwclas | 204 | | 0 | | 23,458 | | 53,315 | | 3,733 | | 7,360 | |
| energy torsion | 204 | | 0 | | 188,172 | | 100,391 | | 7,029 | | 13,859 | |
| backbone vdw | 204 | | 0 | | 467,894 | | 263,471 | | 18,447 | | 36,372 | |
| Entropy sidec | 204 | | 0 | | 0,000 | | 0,000 | | 0,000 | | 0,000 | |
| Entropy manc | 204 | | 0 | | 0,000 | | 0,000 | | 0,000 | | 0,000 | |
| water bonds | 204 | | 0 | | 0,745 | | 1,227 | | 0,0859 | | 0,169 | |
| helix dipole | 204 | | 0 | | 11,769 | | 11,938 | | 0,836 | | 1,648 | |
| loop entropy | 204 | | 0 | | 225,846 | | 162,575 | | 11,383 | | 22,443 | |
| cis bond | 204 | | 0 | | -5,401 | | 4,399 | | 0,308 | | 0,607 | |
| disulfide | 204 | | 0 | | 0,000 | | 0,000 | | 0,000 | | 0,000 | |
| kn electrostatic | 204 | | 0 | | -1,373 | | 5,422 | | 0,380 | | 0,749 | |
| partial covalent | 204 | | 0 | | -0,0111 | | 0,129 | | 0,00905 | | 0,0178 | |
| Energy Ionisation | 204 | | 0 | | -4,628 | | 12,589 | | 0,881 | | 1,738 | |
| Entropy Complex | 204 | | 0 | | 1,215 | | 0,951 | | 0,0665 | | 0,131 | |
| Residue Number | 204 | | 0 | | 321,828 | | 188,517 | | 13,199 | | 26,024 | |
| **Column** | | **Range** | | **Max** | | **Min** | | **Median** | |  | |  |
| DG | | 1308,497 | | 1211,680 | | -96,817 | | -6,636 | |  | |  |
| Back bond | | 679,404 | | -39,679 | | -719,083 | | -190,105 | |  | |  |
| Side Hbond | | 244,070 | | -13,010 | | -257,080 | | -79,474 | |  | |  |
| Energy VdW | | 1244,717 | | -51,073 | | -1295,790 | | -323,783 | |  | |  |
| Electro | | 52,660 | | 0,616 | | -52,044 | | -12,058 | |  | |  |
| Energy SolvP | | 1672,890 | | 1755,170 | | 82,281 | | 426,509 | |  | |  |
| Energy SolvH | | 1639,237 | | -59,073 | | -1698,310 | | -434,223 | |  | |  |
| Energy vdwclas | | 687,601 | | 687,967 | | 0,366 | | 12,827 | |  | |  |
| energy torsion | | 617,679 | | 647,241 | | 29,562 | | 169,096 | |  | |  |
| backbone vdw | | 1959,856 | | 2047,560 | | 87,704 | | 421,152 | |  | |  |
| Entropy sidec | | 0,000 | | 0,000 | | 0,000 | | 0,000 | |  | |  |
| Entropy manc | | 0,000 | | 0,000 | | 0,000 | | 0,000 | |  | |  |
| water bonds | | 7,868 | | 7,868 | | 0,000 | | 0,000 | |  | |  |
| helix dipole | | 99,303 | | 100,285 | | 0,982 | | 7,998 | |  | |  |
| loop entropy | | 1119,122 | | 1158,810 | | 39,688 | | 192,906 | |  | |  |
| cis bond | | 22,265 | | 0,613 | | -21,653 | | -4,189 | |  | |  |
| disulfide | | 0,000 | | 0,000 | | 0,000 | | 0,000 | |  | |  |
| kn electrostatic | | 45,739 | | 0,000 | | -45,739 | | 0,000 | |  | |  |
| partial covalent | | 1,805 | | 0,0121 | | -1,793 | | 0,000 | |  | |  |
| Energy Ionisation | | 119,614 | | 0,000 | | -119,614 | | 0,000 | |  | |  |
| Entropy Complex | | 6,177 | | 6,177 | | 0,000 | | 1,062 | |  | |  |
| Residue Number | | 1168,000 | | 1229,000 | | 61,000 | | 288,500 | |  | |  |

**Non-oscillating genes**

| **Column** | | **Size** | | **Missing** | | **Mean** | | **Std Dev** | **Std. Error** | | | **C.I. of Mean** | |
| --- | --- | --- | --- | --- | --- | --- | --- | --- | --- | --- | --- | --- | --- |
| DG Random | | 95 | | 0 | | 29,837 | | 91,091 | 9,346 | | | 18,556 | |
| Back bond | | 95 | | 0 | | -209,411 | | 121,678 | 12,484 | | | 24,787 | |
| Side Hbond | | 95 | | 0 | | -89,309 | | 52,649 | 5,402 | | | 10,725 | |
| Energy VdW | | 95 | | 0 | | -363,002 | | 211,161 | 21,665 | | | 43,016 | |
| Electro | | 95 | | 0 | | -14,032 | | 10,912 | 1,120 | | | 2,223 | |
| Energy SolvP | | 95 | | 0 | | 486,512 | | 279,323 | 28,658 | | | 56,901 | |
| Energy SolvH | | 95 | | 0 | | -480,658 | | 281,709 | 28,903 | | | 57,387 | |
| Energy vdwclas | | 95 | | 0 | | 24,849 | | 21,934 | 2,250 | | | 4,468 | |
| energy torsion | | 95 | | 0 | | 191,123 | | 109,737 | 11,259 | | | 22,354 | |
| backbone vdw | | 95 | | 0 | | 485,113 | | 258,663 | 26,538 | | | 52,692 | |
| Entropy sidec | | 95 | | 0 | | 0,000 | | 0,000 | 0,000 | | | 0,000 | |
| Entropy manc | | 95 | | 0 | | 0,000 | | 0,000 | 0,000 | | | 0,000 | |
| water bonds | | 95 | | 0 | | 0,693 | | 1,024 | 0,105 | | | 0,209 | |
| helix dipole | | 95 | | 0 | | 12,045 | | 8,639 | 0,886 | | | 1,760 | |
| loop entropy | | 95 | | 0 | | 235,888 | | 161,850 | 16,605 | | | 32,971 | |
| cis bond | | 95 | | 0 | | -5,511 | | 6,128 | 0,629 | | | 1,248 | |
| disulfide | | 95 | | 0 | | 0,000 | | 0,000 | 0,000 | | | 0,000 | |
| kn electrostatic | | 95 | | 0 | | -3,606 | | 7,590 | 0,779 | | | 1,546 | |
| partial covalent | | 95 | | 0 | | 0,000 | | 0,000 | 0,000 | | | 0,000 | |
| Energy Ionisation | | 95 | | 0 | | -6,075 | | 15,550 | 1,595 | | | 3,168 | |
| Entropy Complex | | 95 | | 0 | | 1,108 | | 0,820 | 0,0841 | | | 0,167 | |
| Residue Number | | 95 | | 0 | | 313,274 | | 165,505 | 16,981 | | | 33,715 | |
| **Column** | **Range** | | **Max** | | **Min** | | **Median** | | |  |  | |  |
| DG Random | 433,676 | | 296,712 | | -136,964 | | 14,302 | | |  |  | |  |
| Back bond | 669,688 | | -22,845 | | -692,533 | | -205,919 | | |  |  | |  |
| Side Hbond | 263,163 | | -14,222 | | -277,385 | | -83,165 | | |  |  | |  |
| Energy VdW | 1075,789 | | -67,711 | | -1143,500 | | -323,485 | | |  |  | |  |
| Electro | 59,879 | | 1,262 | | -58,617 | | -11,934 | | |  |  | |  |
| Energy SolvP | 1404,786 | | 1501,620 | | 96,834 | | 436,944 | | |  |  | |  |
| Energy SolvH | 1475,639 | | -84,651 | | -1560,290 | | -436,443 | | |  |  | |  |
| Energy vdwclas | 102,743 | | 104,069 | | 1,326 | | 17,676 | | |  |  | |  |
| energy torsion | 550,605 | | 589,736 | | 39,131 | | 179,802 | | |  |  | |  |
| backbone vdw | 1247,906 | | 1370,030 | | 122,124 | | 447,821 | | |  |  | |  |
| Entropy sidec | 0,000 | | 0,000 | | 0,000 | | 0,000 | | |  |  | |  |
| Entropy manc | 0,000 | | 0,000 | | 0,000 | | 0,000 | | |  |  | |  |
| water bonds | 4,496 | | 4,496 | | 0,000 | | 0,000 | | |  |  | |  |
| helix dipole | 42,818 | | 44,080 | | 1,263 | | 9,800 | | |  |  | |  |
| loop entropy | 976,850 | | 998,816 | | 21,966 | | 203,700 | | |  |  | |  |
| cis bond | 32,781 | | 0,887 | | -31,894 | | -3,741 | | |  |  | |  |
| disulfide | 0,000 | | 0,000 | | 0,000 | | 0,000 | | |  |  | |  |
| kn electrostatic | 38,400 | | 0,000 | | -38,400 | | 0,000 | | |  |  | |  |
| partial covalent | 0,000 | | 0,000 | | 0,000 | | 0,000 | | |  |  | |  |
| Energy Ionisation | 69,607 | | 0,000 | | -69,607 | | 0,000 | | |  |  | |  |
| Entropy Complex | 4,000 | | 4,000 | | 0,000 | | 0,865 | | |  |  | |  |
| Residue Number | 854,000 | | 935,000 | | 81,000 | | 297,000 | | |  |  | |  |
