# Supplementary material for: A Multi-Layered Study on Harmonic Oscillations in Mammalian Genomics and Proteomics
Source: Int J Mol Sci. 2019 Sep 17;20(18):4585. doi: 10.3390/ijms20184585 (PMC6770795; doi:10.3390/ijms20184585)
Supplement: Supplementary file 1 [file ijms-20-04585-s001.zip › TableS8.doc]

| Supplementary Table1. Free energy (ΔG) terms included in the core function of FoldX, the empirical force ﬁeld algorithm aiming to calculate the change of ΔG in kcalmol−1 | | |
| --- | --- | --- |
| Residue Number | Residue Number | Number of residues |
| delta_G | Delta_G | Predicted overall protein stability |
| BackHbond | H-bonds_Backbone | Contribution of backbone Hbonds |
| SideHbond | H-bonds_Sidechain | Contribution of sidechain-sidechain and sidechain-backbone Hbonds |
| Energy_VdW | Energy_VanderWaals | Contribution of the VanderWaals |
| Electro | Electrostat_Int | Electrostatic interactions |
| Energy_SolvP | Penal_PolarGroups | Penalization for burying polar groups |
| Energy_SolvH | Cont_Hydrophobic | Contribution of hydrophobic groups |
| Energy_vdwclash | Penal_VanderWaals | Energy penalization due to Van der Waals’ clashes (interresidue) |
| energy_torsion | VanderWaals_Torsion | Van der Waals’ torsional clashes (intraresidue) |
| backbone_vdwclash | Backbone_VanderWaals | Backbone-backbone Van der Waals |
| Entropy_sidec | Entropy_SideChain | Entropy cost of fixing the side chain |
| Entropy_mainc | Entropy_MainChain | Entropy cost of fixing the main chain |
| water bonds | Water Bonds | Contribution of water bridges |
| helix dipole | Electrostatic_HelixDipole | Electrostatic contribution of the helix dipole |
| loop_entropy | Entropy_Loop | Entropy cost of bringing together two residues of a polymer within a prescribed distance |
| cis_bond | Cost_PeptideBond | Cost of having a cis peptide bond |
| disulfide | Cont_DisulfieBonds | Contribution of disulfide bonds |
| kn electrostatic | Electrostat_Precomplex | Electrostatic interaction between molecules in the precomplex |
| partial covalent interactions | Interaction_BoundMetals | Interactions with bound metals |
| Energy_Ionisation | Energy_Ionisation | Contribution of ionisation energy |
| Entropy Complex | Entropy_Complex | Entropy cost of forming a complex |
|  |  |  |
